# Complete Genome Sequence of a New *Enterococcus faecalis* Bacteriophage, vB_EfaS_IME197

**DOI:** 10.1128/genomeA.00827-16

**Published:** 2016-09-15

**Authors:** Shi Cheng, Shaozhen Xing, Xianglilan Zhang, Guangqian Pei, Xiaoping An, Zhiqiang Mi, Yong Huang, Yigang Tong

**Affiliations:** a Anhui Medical University, Hefei, Anhui, China; b State Key Laboratory of Pathogen and Biosecurity, Beijing Institute of Microbiology and Epidemiology, Fengtai, Beijing, China

## Abstract

We report here the whole-genome sequence of a new *Enterococcus faecalis* phage, vB_EfaS_IME197, which has a linear double-stranded DNA genome of 41,307 bp with 34% G+C content. We describe the main features of the genome of vB_EfaS_IME197.

## GENOME ANNOUNCEMENT

*Enterococcus faecalis* can lead to fatal bacterial infections by its ectopic parasitism, especially after obtaining virulence genes or high levels of antibiotic resistance. These infections include septicemia, urinary tract infections, pyogenic abdominal infections, endocarditis, and others (1–3). In terms of nosocomial infections caused by Gram-positive cocci, *E. faecalis* is second only to *Staphylococcus* (4). The abuse of clinical antibiotics has led to the appearance of a variety of drug-resistant bacteria. Even more frightening is that bacterial genetic material coding for drug resistance genes can be transferred to other pathogens. Recently, lytic bacteriophages or their endolysins have been considered a method to inhibit multidrug-resistant bacteria (5). We isolated a novel bacteriophage, vB_EfaS_IME197, from sewage from the 307th Hospital of the Chinese People’s Liberation Army, Beijing, China, using a strain of *E. faecalis* from the hospital.

Phage genomic DNA was extracted from the stock using the proteinase K-SDS method (6). A 400-bp shotgun library was prepared using the NEBNext Fast DNA library prep set for Ion Torrent (New England BioLabs, USA). Whole-genome sequencing was performed using the Life Technologies Ion Personal Genome Machine sequencer (Ion Torrent). In consequence, 227,855 reads were generated (477× coverage of the genome), and their average length was 294.43 bp. By use of the Roche 454 Newbler version 2.9 assembler, the resulting sequences were *de novo* assembled, and 68,616 reads were mapped onto the complete genome. The complete genome of phage IME197 is a double-stranded linear DNA of 41,307 bp, with 34% G+C content. Running BLASTN with whole genomes showed that it has little similarity to other *E. faecalis* phage genomes, including *Enterococcus* phage EFC-1 (accession no. KJ608188.1), with 42% query cover and 95% identity; *Enterococcus* phage phiEf11 (accession no. GQ452243.1), with 61% query cover and 94% identity; and to prophages in *E. faecalis* strains DENG 1 (accession no. CP004081.1), with 49% query cover and 97% identity and Symbioflor 1 (accession no. HF558530.1), with 63% query cover and 94% identity. The genomic sequence was randomly opened, so we opened the genome upstream of the terminase genes. Genome annotations were performed with Rapid Annotations using Subsystems Technology (7). Of the 67 predicted coding DNA sequences identified, 33 were annotated as known functional genes. The phage genome also contains 2 tRNAs and an integrase gene, which suggest that IME-197 is a lysogenic phage. This genome contains functional genes related to phage packaging (portal protein, capsid and scaffold, and terminase large subunit), regulation and modification (repressor, antirepressor protein, replication initiation, and transcriptional regulator), head morphogenesis (head protein), tail morphogenesis (major tail protein, structural protein, and tail length tape measure protein), host lysis (lysin and holin), and additional functions (integrase, recombination protein, excisionase protein, PcfU, glycerophosphoryl diester phosphodiesterase, abortive infection bacteriophage resistance protein, and choline binding protein D), as well as 34 hypothetical proteins. We picked out the gaps and ran BLASTX for them. The results are that no similar amino acid sequence as the Orf starting at 20,592 bp and 22,317 bp are aligned to hypothetical protein, and the same situation happened to the Orf starting at 23,945 bp and 39,064 bp.

### Accession number(s).

The whole-genome sequence of vB_EfaS_IME197 has been submitted to the National Center for Biotechnology Information GenBank with accession no. KT945994.

## References

[1] Hirsh DC, Martin LD 1983 Rapid detection of *Salmonella* spp. by using Felix-O1 bacteriophage and high-performance liquid chromatography. Appl Environ Microbiol 45:260–264. 6337550 10.1128/aem.45.1.260-264.1983 PMC242263

[2] Kallings LO 1967 Sensitivity of various *Salmonella* strains to Felix O-1 phage. Acta Pathol Microbiol Scand 70:446–454. doi:10.1111/j.1699-0463.1967.tb01312.x. 4867281

[3] Wu L 2009 Study on pathogenicity of putative virulence gene of *Enterococcus* faecium. J Biomed Eng 26:601–605. 19634681

[4] Koskinen T, Lehto H 2012 Prevalence and risk factors for VRE colonisation in a tertiary hospital in Melbourne, Australia: a cross sectional study. Antimicrob Resist Infect Contr 1:1–6. 10.1186/2047-2994-1-31 PMC3523023 23039285

[5] Harper DR, Anderson J, Enright MC 2011 Phage therapy: delivering on the promise. Ther Deliv 2:935–947. doi:10.4155/tde.11.64. 22833904

[6] Sambrook J, Russell DW 2001 Molecular cloning: a laboratory manual, 3rd ed, vol 2 Cold Spring Harbor Laboratory Press, Cold Spring Harbor, NY.

[7] Aziz RK, Bartels D, Best AA, Dejongh M, Disz T, Edwards RA, Formsma K, Gerdes S, Glass EM, Kubal M, Meyer F, Olsen GJ, Olson R, Osterman AL, Overbeek RA, McNeil LK, Paarmann D, Paczian T, Parrello B, Pusch GD 2008 The RAST server: Rapid Annotations using Subsystems Technology. BMC Genomics 9:75. doi:10.1186/1471-2164-9-75. 18261238 PMC2265698

